# Recover then aggregate: unified cross-modal deep clustering with global structural information for single-cell data

**DOI:** 10.1093/bib/bbae485

**Published:** 2024-10-01

**Authors:** Ziyi Wang, Peng Luo, Mingming Xiao, Boyang Wang, Tianyu Liu, Xiangyu Sun

**Affiliations:** Department of Surgical Oncology and General Surgery, First Hospital of China Medical University, Shenyang 110001, PR China; Section of Esophageal and Mediastinal Oncology, Department of Thoracic Surgery, National Cancer Center/National Clinical Research Center for Cancer/Cancer Hospital, Chinese Academy of Medical Sciences and Peking Union Medical College, Beijing 100730, China; Department of Thoracic Surgery, The First Hospital of China Medical University, No.155 North Nanjing Street, Shenyang 110001, People’s Republic of China; Department of Thoracic Surgery, Xinqiao Hospital, Army Medical University, Chongqing 400038, China; Department of Pathology, People’s Hospital of China Medical University (Liaoning Provincial People’s Hospital), Shenyang, Liaoning Province 110015, People’s Republic of China; Electrical and Computer Engineering, University of Illinois at Chicago, Chicago, IL 60607, United States; Computer Science and Engineering, University of California, Riverside, Riverside, CA 92521, United States; Cancer Hospital of China Medical University, Liaoning Cancer Hospital and Institute, Shenyang 110042, Liaoning, China; Cancer Hospital of Dalian University of Technology, Shenyang, Liaoning Province 110042, China

**Keywords:** data recovery, single-cell clustering, cross-modal amalgamation, Laplace matrix

## Abstract

Single-cell cross-modal joint clustering has been extensively utilized to investigate the tumor microenvironment. Although numerous approaches have been suggested, accurate clustering remains the main challenge. First, the gene expression matrix frequently contains numerous missing values due to measurement limitations. The majority of existing clustering methods treat it as a typical multi-modal dataset without further processing. Few methods conduct recovery before clustering and do not sufficiently engage with the underlying research, leading to suboptimal outcomes. Additionally, the existing cross-modal information fusion strategy does not ensure consistency of representations across different modes, potentially leading to the integration of conflicting information, which could degrade performance. To address these challenges, we propose the ’Recover then Aggregate’ strategy and introduce the Unified Cross-Modal Deep Clustering model. Specifically, we have developed a data augmentation technique based on neighborhood similarity, iteratively imposing rank constraints on the Laplacian matrix, thus updating the similarity matrix and recovering dropout events. Concurrently, we integrate cross-modal features and employ contrastive learning to align modality-specific representations with consistent ones, enhancing the effective integration of diverse modal information. Comprehensive experiments on five real-world multi-modal datasets have demonstrated this method’s superior effectiveness in single-cell clustering tasks.

## Introduction

Single-cell sequencing technology, a high-resolution, high-throughput nucleic acid measurement tool, has been widely adopted in life sciences research [1–5]. It precisely captures subtle signals of gene function and variation within cells, acting as a powerful instrument for exploring the complex microenvironments within organisms. Despite the sophistication of this technology, a significant challenge remains in single-cell analysis due to the lack of prior knowledge, particularly in accurately identifying cell identities. Unsupervised clustering algorithms play an important role in single-cell analysis [6–9], as they automatically assign cells into distinct subgroups based on similarities, without the need for external labels. The accuracy of the clustering algorithms is critical, as inaccurate clustering outcomes can lead to a cascade of errors, affecting the precision and reliability of subsequent analyses.

To ensure the accuracy of cell clustering analysis, researchers have dedicated significant efforts in this field [10–13]. Classical algorithms, including $k$-means [14] and spectral clustering [15], were initially applied to single-cell data analysis, marking the nascent stages of work in this area. These traditional methods endeavor to adapt general clustering techniques specifically to the field of single-cell data, achieving limited success. Due to the omission of the unique characteristics of single-cell data, these methods often achieve a suboptimal clustering result. For instance, RaceID incorporates $k$-means clustering into single-cell RNA sequencing datasets to ascertain cluster identities and devises an outlier detection module to enhance the discernment of cellular populations [16]. This algorithm inherits the inherent drawback of $k$-means: sensitivity to the initialization of cluster centers and a propensity to converge to local optima. To address this flaw, SC3 resolves the issue by repeatedly executing $k$-means [17], integrating multiple clustering solutions to achieve a consensus result, thus yielding highly robust clustering outcomes. With the rise of deep learning technologies, a variety of deep clustering algorithms has emerged and rapidly infiltrated the field of single-cell data analysis. These algorithms improve the quality of cell representation through the imposition of specific constraints, effectively leveraging the advancements of representation learning in the single-cell domain to generate improved clustering outcomes. For example, DCA utilizes denoising autoencoders to optimize scRNA-seq data representation [18], employing a zero-inflated negative binomial (ZINB) distribution loss function. In contrast, the scDeepCluster method integrates deep embedded clustering to enhance both the learning of feature representations and the effectiveness of clustering simultaneously [19]. Additionally, single-cell variational inference employs a ZINB to approximate the distribution of expression values, and incorporates the toolkit scvi-tools [20]. Additionally, some researchers have proposed the existence of topological structures among cells, successfully integrating a graph learning model into the single-cell analysis framework, thereby more accurately capturing the interconnected patterns and potential biological relationships between cells. scDeepSort proposes a strategy using gene expression values as edges to construct a cell-gene graph and develops a weighted Graph Neural Network (GNN)-based model for cell annotation [21]. Graph-sc employs a similar component graph strategy [22], utilizing graph autoencoders to conduct clustering analysis on scRNA-seq data. scGAC constructs a cell-cell graph and adopts a self-optimizing approach to simultaneously learn representations and optimize clustering [23], leveraging a graph attention network to extract topological embeddings of cells. scDFC, building upon scGAC, enhances the process by retaining both attribute and structural information, ultimately facilitating the clustering procedure [24]. In recent years, the rapid advancement of sequencing technologies has led to the emergence of multi-modal data, prompting researchers to explore strategies for integrating information from multiple sources to analyze cell populations from a broader perspective. Consequently, multi-modal clustering algorithms have become a research frontier [25–28], aiming to synthesize different types of biological data to deepen our understanding of cellular heterogeneity and functional networks.

Despite significant advancements in the field of clustering algorithms, existing cross-modal clustering methods exhibit two major limitations. First, these algorithms frequently overlook the prevalent issue of missing signals in single-cell data, erroneously treating these missing values as zeroes. This omission not only results in the loss of critical modal expressions in the original data but, in extreme cases, might even lead to fundamentally flawed biological conclusions [29, 30]. A minority of algorithms focus on the recovery of single-cell data [31–34]; however, they concentrate solely on data recovery, just performing simplistic clustering analysis on the imputed numerical matrix. They consider data recovery as an independent data preprocessing approach, separating data recovery and data aggregation into two distinct steps, thus hindering the model’s ability to generate cluster-friendly cellular representations. Consequently, these recovery methods often yield suboptimal clustering outcomes, making it urgent to develop a clustering algorithm that can effectively handle missing data.

Cross-modal aggregation poses another significant challenge. As the application of multi-modal data becomes increasingly prevalent, numerous cross-modal deep clustering algorithms have emerged [35–38]. Such methods utilize specific encoder networks to compress raw data into low-dimensional feature spaces and subsequently fuse these representations. However, as these representations are not aligned within the feature space, aggregation is often compromised by modal-specific private information. Some algorithms align data representations using Kullback–Leibler divergence and pseudo-labels before aggregation [39]. However, these sample-level alignment algorithms can potentially be disrupted by conflict cluster information since clustering assignments in one modality may differ from those in another. Additionally, most aggregation methods often overlook differences in data quality across modalities, treating all modal representations as equally important. This approach unfairly disadvantages modalities with superior data quality and results in suboptimal clustering outcomes. In summary, existing cross-modal aggregation methods are limited in accuracy and effectiveness, with significant potential for improvement.

To address the aforementioned challenges, we have devised a novel ’recover-then-aggregate’ deep clustering framework tailored for single-cell data with missing values. This model comprises three pivotal components: (1) A discriminative data recovery module assesses the confidence of zero values to determine whether to perform data recovery via Non-negative Matrix Factorization, thus circumventing the introduction of extraneous noise from over-recovery. (2) A cross-view aggregation module that constructs a global structural relationship matrix based on an attention mechanism, assigning weights to each independent mode during the aggregation process, thereby facilitating more efficient data integration. (3) A contrastive learning optimization module that employs contrastive loss to draw samples belonging to the same cluster closer together, jointly optimizing it with reconstruction loss to yield high-quality cell representations. To summarize, our contributions are three-fold:

- **Problem:** we propose a pioneering recovery-then-aggregate clustering framework to fully aggregate cross-modal information through structural alignment. To the best of our knowledge, this is the first attempt to integrate data recovery and aggregation into a unified framework and apply structure-guided contrastive loss from different modalities for cross-modal information aggregation.
- **Algorithm:** we propose a discriminative multi-modal data recovery method and structure-guided aggregation approaches, which improve the quality of single-cell data and achieve better fusion.
- **Evaluation:** we have conducted extensive experiments to validate the effectiveness of Unified Cross-Modal Deep Clustering (UCMDC) with state-of-the-art (SOTA) single-cell clustering methods.

## Methods

In this section, we will delineate the UCMDC model in the following sequence: Problem Description, Data Recovery, Cross-Modal Aggregation, Cell Representation Optimization, and Model Evaluation.

### Problem description

**Figure 1:**
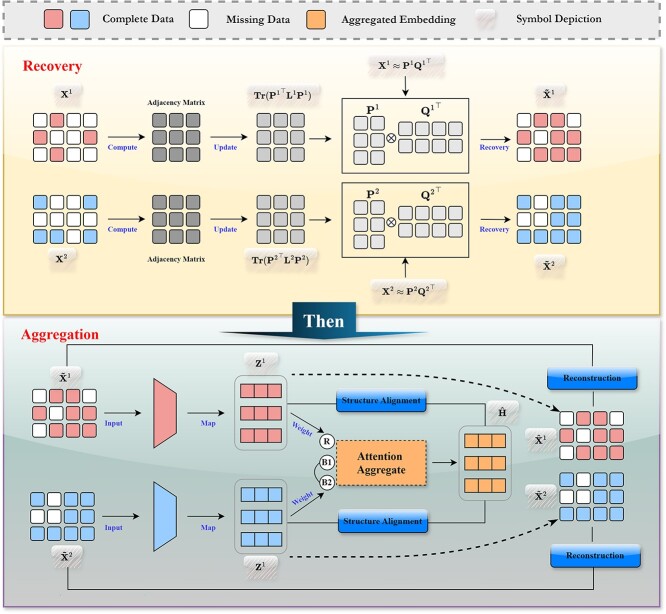
The framework of the UCMDC. It is categorized into two main modules: the data recovery module and the cross-modal aggregation and optimization module. The data recovery module employs the trace constraint of matrices to update the similarity between cells, facilitating data recovery through non-negative matrix factorization, while the cross-modal aggregation optimization module uses an attention mechanism to aggregate representations from different modalities and refines these with a global structural relationship matrix. Ultimately, contrastive learning is used to bridge the gap between modality-specific representations and consistent representations, with the goal of achieving high-quality cell representations.

The detailed procedure for UCMDC is illustrated in Fig. 1. For clarity, single-cell multi-modal data is mathematically represented as follows: 


(1) \begin{align*}& \begin{aligned} \mathbf{X}^{m}=\{\mathbf{x}_{1}^{m};...;\mathbf{x}_{N}^{m}\}\in \mathbb{R}^{N \times D_{m}}, \end{aligned}\end{align*}

where $\mathbf{X}^{m}$ represents the data of the $m$th modality, while $D_{m}$ represents the feature dimension in the $m$th modality. The dataset comprises N samples across a total of M modalities.

### Data recovery

It is common for single-cell data to contain missing values. The reasons for missing data arise from various biological and technical sources. For instance, biological non-expression and the omission of signal detection during sequencing both often result in zero values. Indiscriminate treatment of these zeros can introduce extraneous noise and compromise the accuracy of the analysis. To address this issue, we propose a data augmentation technique that leverages neighborhood similarity, iteratively imposing rank constraints on the Laplacian matrix to update the similarity matrix and recover dropout events. This strategy aligns with our real-world intuition that utilizing neighboring information to infer and fill gaps when data signals are missing facilitates data recovery. The process initiates with the concept of recovery confidence. This metric aids in determining which zero values are suitable for recovery. The definition of recovery confidence is as follows: 


(2) \begin{align*}& \begin{aligned} c_{j}^{(k)} & = \frac{\left(1-a_{j}^{(k)}\right) b_{j}^{(k)}}{\left(1-a_{j}^{(k)}\right) b_{j}^{(k)}+a_{j}^{(k)} \mathcal{V}_{j}^{2(k)}}, \end{aligned}\end{align*}

where $a_{j}^{(k)}$, $b_{j}^{(k)}$, and $ \mathcal{V}_{j}^{2(k)}$ signify the zero expression rate, the average expression level, and the variance of expression values for gene $j$ within its respective $k$th cluster. Clearly, $c_{j}^{(k)}$ computes the normalized probability values, ranging between 0 and 1 as $a_{j}^{(k)}$ and other parameters vary.

Next, we enter the critical phase of determining whether to perform data recovery. For this purpose, we constructed a recovery guidance matrix $\mathbf{G}$ with dimensions identical to those of the original input matrix $\mathbf{X}$. The elements of $\mathbf{G}$ are binary, consisting only of zeros and ones. A recovery threshold $\mathcal{T}$ was established to regulate the extent of recovery. Elements failing to meet this threshold are marked with one in matrix $\mathbf{G}$, thus enabling selective recovery. The definition of matrix $\mathbf{G}$ is as follows: 


(3) \begin{align*}& \begin{aligned} G_{ij} & = \left\{\begin{array}{ll} 1 & \textrm{ if } X_{ij}>0, \\ 1 & \textrm{ if } c_{j}^{(k)}<\mathcal{T},\\ 0 & \text{ otherwise. } \end{array}\right. \end{aligned}\end{align*}

where the element $X_{ij}$ in the matrix $\mathbf{X}$ denotes the value at the $i$th row and $j$th column of the expression matrix, and the corresponding element $G_{ij}$ in the guidance matrix $\mathbf{G}$ determines whether data recovery should be performed at that location. With the guidance matrix obtained, we then formulated the optimization objective. Specifically, non-negative matrix factorization is utilized to identify and restore missing values in multi-modal, non-negative single-cell data. The mathematical formulation is as follows: 


(4) \begin{align*}& \begin{aligned} \min_{\substack{\mathbf{P}_{i} \geq 0, \forall m,\mathbf{Q} \geq 0}} \sum_{m=1}^{M} \left\| \mathbf{G}^{m} \odot (\mathbf{X}^{m} - \mathbf{P}^{m} {\mathbf{Q}^{m}}^{\top}) \right\|_{F}^{2}, \end{aligned}\end{align*}

where $\mathbf{P}^{m}$ denotes the $m$th latent features of the cells, and $\mathbf{Q}^{m}$ denotes the $m$th latent features of the genes. The symbol $\odot $ represents the Hadamard product operation, defined as the element-wise multiplication of matrices. Moreover, it is assumed that all cells $\{p_{1}, p_{2}, \ldots , p_{n}\}$ can be connected to $p_{i}$ as a neighbor with probability $s_{ij}$, where the similarity among cells can be computed by optimizing the following probabilistic neighbors objective: 


(5) \begin{align*}& \begin{aligned} \min\sum_{m=1}^{M} \sum_{i,j} s_{ij}^{(m)} \left\| p_{i}^{(m)} - p_{j}^{(m)} \right\|^{2}. \end{aligned}\end{align*}

According to the properties of matrix trace, Equation (5) can be equivalently substituted by its trace constraint as follows: 


(6) \begin{align*}& \begin{aligned} \min\sum_{m=1}^{M} \mathrm{Tr} \left( (\mathbf{P}^{m})^{\top} \mathbf{L}^{m} \mathbf{P}^{m} \right), \end{aligned}\end{align*}

where $\mathbf{L}^{m}$ represents the Laplacian matrix for the $m$th modality. We combine Equations (4) and (6), along with an additional regularization term, to construct the total optimization objective, which can be mathematically expressed as follows: 


(7) \begin{align*}& \begin{aligned} &\min_{\substack{\mathbf{P}_{i} \geq 0, \forall m,\mathbf{Q} \geq 0}} \sum_{m=1}^{M} \left\| \mathbf{G}^{m} \odot (\mathbf{X}^{m} - \mathbf{P}^{m} {\mathbf{Q}^{m}}^{\top}) \right\|_{F}^{2} \\ &+ \lambda_{1} \left( \| \mathbf{P}^{m} \|_{F}^{2} + \| \mathbf{Q}^{m} \|_{F}^{2} \right) + \lambda_{2} \operatorname{Tr} \left( {\mathbf{P}^{m}}^{\top} \mathbf{L}^{m} \mathbf{P}^{m} \right), \end{aligned}\end{align*}

where $\lambda _{1}$ and $\lambda _{2}$ are the regularization parameters. To solve the optimization problem described above, the objective function is abbreviated as $ \textrm{Obj} $. By setting $\frac{\partial \textrm{Obj}}{\partial P} = 0$ and $\frac{\partial \textrm{Obj}}{\partial Q} = 0$, the following update rules are derived: 


(8) \begin{align*} & \mathbf{P}^{m} = \mathbf{P}^{m} \odot \frac{\left((\mathbf{G}^{m} \odot \mathbf{X}^{m}) \mathbf{Q}^{m} + \lambda_{2} \mathbf{D}^{m^{-1/2}} \mathbf{S}^{m} \mathbf{D}^{m^{-1/2}}\right)}{\left((\mathbf{G}^{m} \odot (\mathbf{P}^{m} (\mathbf{Q}^{m})^{\top})) \mathbf{Q}^{m} + \lambda_{1} \mathbf{P}^{m} + \lambda_{2} \mathbf{D}^{m^{-1/2}} \mathbf{D}^{m} \mathbf{D}^{m^{-1/2}}\right),} \end{align*}

(9) \begin{align*} & \mathbf{Q}^{m} = \mathbf{Q}^{m} \odot \frac{\left(\mathbf{P}^{m^{\top}} (\mathbf{G}^{m} \odot \mathbf{X}^{m})\right)}{(\mathbf{P}^{m^{\top}} (\mathbf{G}^{m} \odot (\mathbf{P}^{m} \mathbf{Q}^{m^{\top}})) + \lambda_{1} \mathbf{Q}^{m})}, \end{align*}

where Equations (8) and (9) are used to compute matrices $ \mathbf{P}^{m} $ and $ \mathbf{Q}^{m} $, respectively, until convergence. The learned matrices $ \mathbf{P}^{m} $ and $ \mathbf{Q}^{m} $ are then utilized for data recovery, with the recovered data $\tilde{\mathbf{X}}^{m}$ being computed as follows: 


(10) \begin{align*}& \tilde{\mathbf{X}}^{m} = (1 - \mathbf{G}^{m}) \odot (\mathbf{P}^{m} \mathbf{Q}^{m^{\top}}) + \mathbf{X}^{m}.\end{align*}

### Cross-modal aggregation

Upon completion of the data recovery phase, the next objective is to aggregate the recovered multi-modal data. For the $ m $th modality, let $ f_{\theta ^{m}}^{m} (\cdot ) $ denote the encoder’s nonlinear function. This function compresses the high-dimensional features into a compact, low-dimensional representation, as follows: 


(11) \begin{align*}& \begin{aligned} \mathbf{z}_{i}^{m}=f_{\theta^{m}}^{m}\left(\tilde{\mathbf{x}}_{i}^{m}\right). \end{aligned}\end{align*}

After compression, the modality-specific representations $ \mathbf{Z}^{m} $ are concatenated to form $ \mathbf{Z} $, aiming to thoroughly explore the global structural relationships among the samples. The mathematical formulation is expressed as follows: 


(12) \begin{align*}& \begin{aligned} \mathbf{Z} =\left[\mathbf{Z}^{1}, \mathbf{Z}^{2}, \ldots, \mathbf{Z}^{M}\right]. \end{aligned}\end{align*}

Inspired by the widely adopted transformer architecture [40, 41], we aggregate the representations of different modalities using attention mechanisms. Features from various modalities are uniformly mapped into a new feature space, represented by the matrix $ \mathbf{R} $. The mathematical formulation can be described as follows: 


(13) \begin{align*}& \left[{\begin{array}{@{}c@{}} {{{\textbf{R}}_{1:}}}\\{{{\textbf{R}}_{2:}}}\\ \vdots \\{{{\textbf{R}}_{n:}}} \end{array}} \right] = \left[{\begin{array}{@{}cccc@{}} {{\textbf{z}}_{1}^{1}}&{{\textbf{z}}_{1}^{2}}& \cdots &{{\textbf{z}}_{1}^{M}}\\{{\textbf{z}}_{2}^{1}}&{{\textbf{z}}_{2}^{2}}& \cdots &{{\textbf{z}}_{2}^{M}}\\ \vdots & \vdots & \ddots & \vdots \\{{\textbf{z}}_{n}^{1}}&{{\textbf{z}}_{n}^{2}}& \cdots &{{\textbf{z}}_{n}^{M}} \end{array}} \right]\left[{\begin{array}{@{}c@{}} {{{\textbf{W}}_{R1:}}}\\{{{\textbf{W}}_{R2:}}}\\ \vdots \\{{{\textbf{W}}_{RM:}}} \end{array}} \right]\end{align*}

Similarly, we perform additional feature mapping twice, which can be expressed as follows: 


(14) \begin{align*}& \begin{aligned} \mathbf{B}_{1} =\mathbf{Z}\mathbf{W}_{1}; \mathbf{B}_{2} =\mathbf{Z}\mathbf{W}_{2}; \end{aligned}\end{align*}

where $\mathbf{W}_{1}$ and $\mathbf{W}_{2}$ are two mapping matrices, $\mathbf{B}_{1}$ and $\mathbf{B}_{2}$ are feature matrices used to calculate the global structural relationships $\mathbf{S}$ as follows: 


(15) \begin{align*}& \begin{aligned} \mathbf{S}=\operatorname{softmax}\left(\frac{\mathbf{B}_{1}\mathbf{B}_{2}^{T}}{\sqrt{d}}\right). \end{aligned}\end{align*}

The previously obtained mapping matrix $\mathbf{R}$ is fine-tuned by the global structural relationship matrix $\mathbf{S}$, suggesting that individual cell representations can be enhanced by other highly related samples, with different weights assigned accordingly: 


(16) \begin{align*}& \begin{aligned} \widehat{{{\textbf{z}}_{i}}} = \sum\limits_{j = 1}^{n} {{{\textbf{S}}_{ij}}{\textbf{R}}_{j:}}, \end{aligned}\end{align*}

where $\widehat{{{\textbf{z}}_{i}}}$ denotes the aggregated representation, learned from the concatenation of features across all views, this representation often contains redundant information. To prevent network degradation, we apply a fine-tuning approach to $\mathbf{Z}$, as outlined below: 


(17) \begin{align*}& \begin{array}{l} \widehat{\textbf{H}} = {\textbf{W}_{3}} (\textbf{Z}+\hat{\textbf{Z}}) + {b_{3}} \end{array}\end{align*}

where $\widehat{\textbf{H}}$ denotes the final aggregated cell representation. 



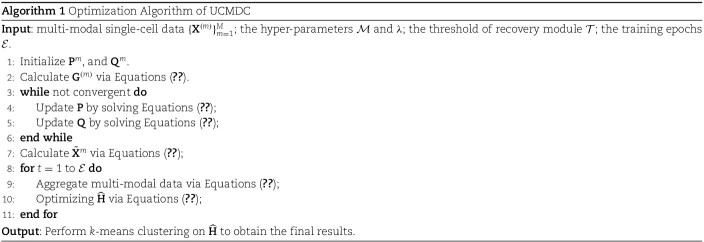



### Cell representation optimization

With the aggregated representations of cell data across different modalities obtained, next, we elaborate on the optimization of these cell representations. The completed optimization process is outlined as shown in Algorithm (1).

$\mathbf{Reconstruction}$ $\mathbf{Loss}$ . Based on the practices of previous research, we train the network to map compressed features back to the original feature space, ensuring that the reconstructed features remain consistent with the original features. This approach boosts the network’s ability to learn effective features, and its mathematical formulation is as follows: 


(18) \begin{align*}& \begin{aligned} \hat{\mathbf{x}}_{i}^{m}=g_{\eta^{m}}^{m}\left(\mathbf{z}_{i}^{m}\right)=g_{\eta^{m}}^{m}\left(f_{\theta^{m}}^{m}\left(\tilde{\mathbf{x}}_{i}^{m}\right)\right) \end{aligned}\end{align*}

where $ g_{\eta ^{m}}^{m} $ represents the mapping network of the decoder, we defined the reconstruction loss for the model as follows: 


(19) \begin{align*}& \begin{aligned} \mathcal{L}_{\mathrm{r}}=\sum_{m=1}^{M}\mathcal{L}_{\mathrm{r}}^{m}=\sum_{m=1}^{M}\left\|\tilde{\mathbf{X}}^{m}-\hat{\mathbf{X}}^{m}\right\|_{2}^{2}\\ =\sum_{m=1}^{M}\sum_{i=1}^{n}\left\|\tilde{\mathbf{x}_{i}}^{m}-g_{\eta^{m}}^{m}\left(\mathbf{z}_{i}^{m}\right)\right\|_{2}^{2} \end{aligned}\end{align*}

$\mathbf{Clustering}$ $\mathbf{Loss}$ . We posit that the consensus representation $\widehat{\textbf{H}}$ and the view-specific representation $\mathbf{H}^{m}$, if both derived from the same cluster, should be closely aligned. To achieve this, we apply contrastive learning to this domain. Specifically, we initially compute the similarity between the consensus representation $\widehat{\textbf{H}}$ and the view-specific representation $\mathbf{H}^{m}$ as follows: 


(20) \begin{align*}& \begin{aligned} D\left({\hat{\mathbf{H}}}_{i:}, \mathbf{H}_{i}^{m}\right)=\frac{{\hat{\mathbf{H}}}_{i:}^{\top}\mathbf{H}_{i:}^{m}}{\left\|{\hat{\mathbf{H}}}_{i:}\right\|\left\|\mathbf{H}^{m}_{i:}\right\|}, \end{aligned}\end{align*}

where the contrastive loss is defined as follows: 


(21) \begin{align*}& \begin{aligned} \mathcal{L}_{\mathrm{c}}=-\frac{1}{2 N} \sum_{i=1}^{N} \sum_{m=1}^{M} \log \frac{e^{\operatorname{D}\left({\hat{\mathbf{H}}}_{i:}, \mathbf{H}_{i:}^{m}\right) / \mathcal{M}}}{\sum_{j=1}^{N} e^{(1-S_{ij})\operatorname{D}\left({\hat{\mathbf{H}}}_{i:}, \mathbf{H}_{j:}^{m}\right) / \mathcal{M}}-e^{1 / \mathcal{M}}} \end{aligned}\end{align*}

where $\mathcal{M}$ represents the temperature parameter and $\mathbf{S}$ denotes the global structural relationship matrix. A clear negative correlation exists between $ S_{ij} $ and $ D\left (\mathbf{\hat{H}}_{i:}, \mathbf{H}_{i}^{m}\right )$, if $ S_{ij} $ is low then $ D\left ( {\hat{\mathbf{H}}}_{i:}, \mathbf{H}_{i}^{m}\right )$ is high. Fundamentally, when the structural relationship $S_{ij}$ between the ith and jth samples is low, indicating they are not from the same cluster, their representations exhibit inconsistency.

The proposed UCMDC model is jointly optimized for both reconstruction and clustering losses. To optimally exploit the synergy between these two aspects, a hyperparameter $\lambda $ is introduced as a balancing coefficient to regulate their interplay. The total loss is defined as follows: 


(22) \begin{align*}& \begin{aligned} \mathcal{L} = \mathcal{L}_{r} + \lambda \mathcal{L}_{c} \end{aligned}\end{align*}

### Model evaluation

We employ three well-known clustering evaluation metrics in this research: Accuracy (ACC), Normalized Mutual Information (NMI), and Adjusted Rand Index (ARI). Each of these metrics reflects a distinct aspect of a model’s performance. By analyzing these metrics, we can comprehensively assess the model’s effectiveness. ACC is the most straightforward metric for classification performance, measuring the correctness of a model’s predictions, which is defined as below: 


(23) \begin{align*}& \mathrm{ACC}=\frac{\sum_{i=1}^{n}I\left(y_{i}=\hat{y}_{i}\right)}n.\end{align*}

NMI is a clustering evaluation metric based on information theory, evaluating the degree of information sharing between the clustering results and the true data labels: 


(24) \begin{align*}& \mathrm{NMI}=\frac{\sum_{i=1}^{N_{\hat{Y}}}\sum_{j=1}^{N_{Y}}|\hat{Y}_{i}\bigcap Y_{j}|log\frac{N\times|\hat{Y}_{i}\bigcap Y_{j}|}{\hat{Y}_{i}\times|Y_{j}|}}{max(-\sum_{i=1}^{N_{\hat{Y}}}|\hat{Y}_{i}|log\frac{\hat{Y}_{i}}{N},-\sum_{j=1}^{N_{Y}}|Y_{j}|log\frac{|Y_{j}|}{N})}.\end{align*}

ARI is a metric that quantifies the similarity between two data partitions, ensuring a fairer comparison across clusters of varying sizes: 


(25) \begin{align*} \mathrm{ARI}=\ &\frac{\sum_{i=1}^{N_{\hat{Y}}}\sum_{j=1}^{N_{Y}}\left(\begin{array}{c}|\hat{Y}_{i}\cap Y_{j}|\\2\end{array}\right)}{\frac{1}{2}\left[\sum_{i=1}^{N_{\hat{Y}}}\left(\begin{array}{c}|\hat{Y}_{i}|\\2\end{array}\right)+\sum_{j=1}^{N_{Y}}\left(\begin{array}{c}|Y_{j}|\\2\end{array}\right)\right]}\nonumber \\ &\left.\frac{-\left[\sum_{i=1}^{N_{\hat{Y}}}\left(\begin{array}{c}|\hat{Y_{i}}|\\2\end{array}\right.\right)\sum_{j=1}^{N_{Y}}\left(\begin{array}{c}|Y_{j}|\\2\end{array}\right]/\left(\begin{array}{c}N\\2\end{array}\right)}{-\left[\sum_{i=1}^{N_{\hat{Y}}}\left(\begin{array}{c}|\hat{Y_{i}}|\\2\end{array}\right)\sum_{j=1}^{N_{Y}}\left(\begin{array}{c}|Y_{j}|\\2\end{array}\right)\right]/\left(\begin{array}{c}N\\2\end{array}\right)}.\right . \end{align*}

## Experiments

We conducted a series of experiments to evaluate the model’s performance. Specifically, our research addressed the following five scientific questions:

**Q1.** Does the data recovery module effectively handle missing values?

**Q2.** Does the recovered data enhance clustering performance?

**Q3.** Is the UCMDC method superior in deep single-cell clustering tasks?

**Q4.** Does the aggregation and contrastive learning module enhance the optimization of cell representations?

**Q5.** What is the impact of hyper-parameters on the performance of UCMDC?

### Experimental settings

#### Datasets and baseline methods

In this work, we utilized four single-cell multi-modal datasets from various sources, which might have undergone different preprocessing methods. The uniform treatment protocol for these datasets is as follows: If a dataset has already been preprocessed by its original authors, we refrain from further processing. For unprocessed datasets, we employ the Scanpy package to exclude cells or genes with zero counts for quality control. We consistently set the number of highly variable genes to 2000. The sources of these datasets include Pbmc and Smage from the 10X Genomics website (https://www.10xgenomics.com/resources/datasets), Stuart from (https://www.ncbi.nlm.nih.gov/geo/query/acc.cgi?accGSE128639), and Gayoso from (https://github.com/YosefLab/totalVI_reproducibility). The details have been displayed in Table 1

**Table 1:** Single-cell multi-modal datasets involved in this study

Dataset	Cell	Modality	Category	Fea1	Fea2
Stuart	1728	2	5	1000	25
Pbmc	3762	2	16	1000	49
Gayoso	6018	2	10	1000	112
Smage	2585	2	14	2000	2000

Along with our proposed UCMDC, we run eight SOTA multi-modal clustering methods for comparison, including $k$-means [14], spectral clustering [15], Fast Multi-View Clustering Via Ensembles: Towards Scalability, Superiority, and Simplicity (FastMICE) [42], Structured Graph Learning for Scalable Subspace Clustering: From Single View to Multiview (MSGL) [43], Parameter-Free Auto-Weighted Multiple Graph Learning: A Framework for Multiview Clustering and Semi-Supervised Classification (AMGL) [44], Deep cross-omics cycle attention model for joint analysis of single-cell multi-omics data (DCCA) [45], Effective multi-modal clustering method via skip aggregation network for parallel scRNA-seq and scATAC-seq data (scEMC) [46], and Deep-joint-learning analysis model of single cell transcriptome and open chromatin accessibility data (scMVAE) [47].

#### Model implementation

The experiments are implemented on an Ubuntu server equipped with an Intel Core i5-12600KF CPU, 64GB of DDR4 RAM, and an NVIDIA RTX 4060Ti graphics card. The server runs Ubuntu 22.04 LTS, and the algorithm was implemented in Python 3.7 using the Pytorch deep learning framework, version 1.13.1. The encoding layers were configured as (512, 64), with a bottleneck layer size of 64. The model underwent a pretraining phase of 200 epochs, followed by a training phase that also lasted 200 epochs. The learning rate was set at 0.0005. The imputation code is available at https://github.com/ZyiWang/UCMDC.

### Effectiveness of data recovery (Q1)

**Figure 2:**
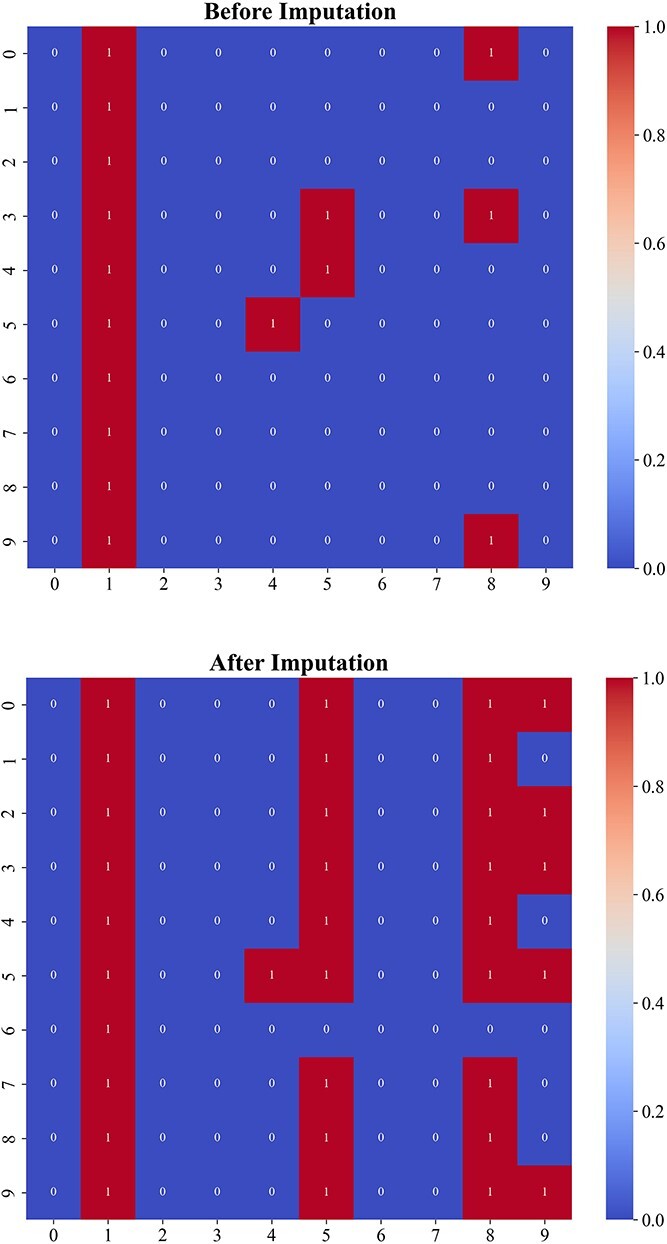
Comparison of data before and after the execution of the data recovery module using dataset Gayoso. Values in areas of missing data are assigned a 0 and are highlighted in blue, whereas values in areas without missing data are assigned a 1 and are highlighted in red.

**Figure 3:**
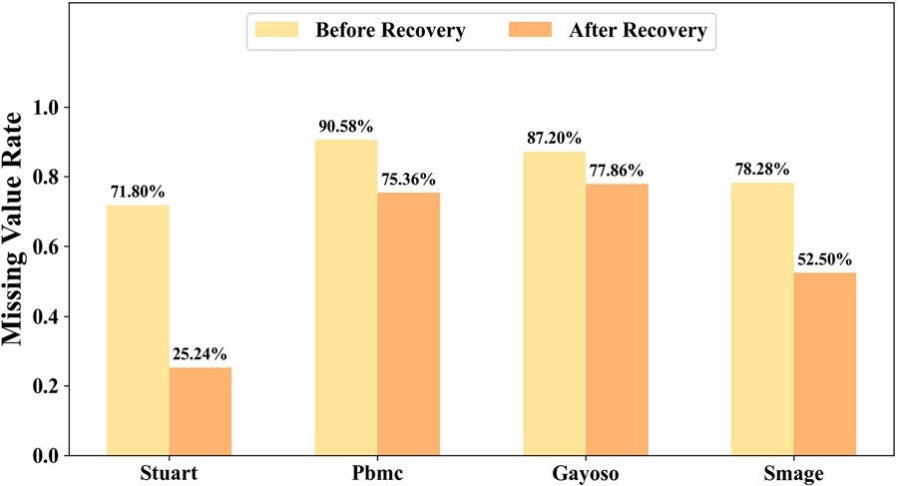
Comparison of the Missing Value Rate before and after the implementation of the data recovery process. The Missing Value Rate is defined as the proportion of zero values relative to the total number of values.

A core component of the UCMDC model is the discriminative data recovery module. In the field of single-cell cluster analysis, the integrity of data is crucial. Defective data can lead to suboptimal outcomes, and excessive interpolation may introduce additional noise. Therefore, we designed a selective data recovery module that assesses the need for data recovery based on the confidence levels associated with zero values. Specifically, we visualized the data before and after recovery, as illustrated in Fig. 2. Data without missing values is represented by a ’1’ and marked in red, while missing data is denoted by a ’0’ and highlighted in blue. Comparing the results before and after data recovery, we can clearly observe that our data recovery module effectively resolved the missing values. Following data recovery, the number of red squares significantly increased, indicating effective recovery. Furthermore, to quantitatively demonstrate the recovery, we presented the proportion of missing values before and after recovery, as shown in Fig. 3. It is evident from the figure that the proportion of missing data significantly decreased after recovery. These results confirm that our data recovery module effectively addresses the common issue of missingness in single-cell data.

### Ablation study of data recovery module (Q2)

In the previous section, we outlined the remarkable efficacy of the data recovery module in handling missing data. However, one key question remains unresolved: Are the recovered data genuinely effective, and do they substantially enhance clustering results? To address this issue, we meticulously designed and conducted an exhaustive ablation experiment to elucidate the impact of the data recovery module on enhancing clustering performance.

Specifically, we compared the performance of the original UCMDC model against a carefully designed variant. In this variant, all modules remained unchanged, except the single-cell matrix with recovered data was replaced by the unrecovered data. The experimental results are systematically outlined in Table 2. By analyzing these numerical differences, we observed that with the support of recovered data, the UCMDC model achieved performance improvements across multiple evaluation metrics, including 19.10, 9.20, 19.46, 7.12, 14.89, 0.29, 7.04, 1.68, 35.78, 4.59, 26.45, and 7.70%. These significant improvements illustrate not only the successful recovery of a substantial amount of data by the UCMDC model but also the considerable effectiveness of this recovery in positively impacting clustering performance. In summary, the results of this ablation experiment underscore its crucial role in enhancing the performance of clustering analyses.

**Table 2:** Comparison of clustering performance with and without the data recovery module.

Metric	Dataset	UCMDC w/o Recovery	UCMDC
ACC	Stuart	0.7506	**0.9416**
	Pbmc	0.6377	**0.7297**
	Gayoso	0.5224	**0.7170**
	Smage	0.6499	**0.7211**
NMI	Stuart	0.6830	**0.8319**
	Pbmc	0.6771	**0.6800**
	Gayoso	0.6075	**0.6779**
	Smage	0.5935	**0.6103**
ARI	Stuart	0.5305	**0.8883**
	Pbmc	0.5454	**0.5913**
	Gayoso	0.4666	**0.7311**
	Smage	0.5476	**0.6246**

### Clustering comparison (Q3)

In the single-cell clustering task, we meticulously designed and executed a comprehensive series of experiments to compare the clustering performance of our proposed UCMDC model with eight baseline methods. The research results are unequivocal and uniform, demonstrating the superior performance of the UCMDC model across all evaluation metrics, as shown in Table 3. In the table, the highest results of the UCMDC model are bold marked to emphasize its exceptional performance, while the second-highest results are distinctly underlined to facilitate easy identification.

**Table 3:** Comparison of UCMDC with eight benchmark methods across four datasets, evaluating ACC, NMI, and ARI scores. The highest-ranked results are highlighted in bold, while the second-highest are underlined to provide emphasis.

Dataset	Metric	Kmeans	Spectual	FastMICE	MSGL	AMGL	DCCA	scEMC	scMVAE	OMVFC	UCMDC
Stuart	ACC	0.6389	0.7240	0.7862	0.7062	0.2168	0.7130	0.6458	0.6542	0.5938	**0.9416**
	NMI	0.6726	0.6478	0.7560	0.6905	0.3200	0.6035	0.6645	0.6819	0.4477	**0.8319**
	ARI	0.4477	0.4450	0.5877	0.4989	0.0004	0.4934	0.4354	0.4562	0.2193	**0.8883**
Pbmc	ACC	0.5811	0.6146	0.6623	0.6539	0.0850	0.6449	0.5534	0.6135	0.4556	**0.7297**
	NMI	0.6095	0.6255	0.7019	0.6658	0.0130	**0.7025**	0.6718	0.6850	0.5791	0.6800
	ARI	0.4349	0.4301	0.5613	0.5312	0.0001	0.5510	0.4159	0.5195	0.3670	**0.5913**
Gayoso	ACC	0.5150	0.6143	0.4923	0.5173	0.1165	0.5243	0.5452	0.5288	0.5035	**0.7170**
	NMI	0.6622	**0.6886**	0.6525	0.6027	0.0066	0.6649	0.5295	0.6678	0.4585	0.6779
	ARI	0.4338	0.5387	0.4150	0.4099	0.0005	0.4550	0.2743	0.4561	0.3452	**0.7311**
Smage	ACC	0.4418	0.5176	0.4793	0.5553	0.0932	0.4878	0.6050	0.4569	0.6468	**0.7211**
	NMI	0.5498	0.5508	0.5601	0.5211	0.0183	0.5334	**0.6122**	0.5394	0.5235	0.6103
	ARI	0.3434	0.3589	0.3244	0.3877	0.0002	0.3817	0.4352	0.3008	0.5130	**0.6246**
Smage-10k	ACC	0.5594	0.6525	0.5053	0.5308	0.0936	0.4721	0.6029	0.4298	0.7366	**0.7473**
	NMI	0.5861	0.5679	0.5725	0.5468	0.0034	0.5463	**0.6173**	0.5480	0.5527	0.5881
	ARI	0.4650	0.4982	0.3738	0.4123	0.0001	0.3881	0.5265	0.2959	0.6199	**0.6691**

From the results, the UCMDC model ranked in the top two in almost all comparative analyses, with only one instance where it failed to achieve the top ranks. More notably, in 12 performance comparisons, the UCMD model achieved the best performance in 9 instances, with significant performance improvements compared to the runner-up amounting to 19.77, 10.04, 51.15, 10.18, 7.31, 16.72, 35.72, 19.19, and 43.52%. These results unequivocally demonstrate the strong advantages of the UCMDC model. Furthermore, the data presented in the table also highlight an interesting phenomenon: the FastMICE model frequently secured the second position, clearly evidencing the effectiveness of multi-level fusion strategies in multi-modal clustering. However, the performance of the AMGL model was comparatively weak, potentially due to its design of modeling data as graphs. Any bias in graph construction can severely impact the model’s performance. In summary, the UCMDC model not only showed unrivaled dominance in key index such as ACC, NMI, and ARI, but also established its powerful potential as a universally applicable clustering method amidst the complexity of biological environments and the diversity of single-cell data distributions. Despite numerous challenges, the UCMDC model displayed consistently excellent performance in almost all evaluation tasks, clearly establishing a solid foundation for its future applications.

### Ablation study of the aggregation and optimization modules (Q4)

In the design of the UCMDC model, beyond the core data recovery module, we strategically integrated a cross-modal aggregation module and a contrastive learning module. To ensure that these carefully designed modules fulfilled their intended functions, we conducted additional ablation experiments to validate their effectiveness and necessity. Specifically, we constructed two variants of the UCMDC model by eliminating the cross-modal aggregation module (CA module) and the contrastive learning module (CL module) independently, to enable an in-depth performance comparison.

**Table 4:** Investigation of the confidence threshold $\mathcal{T}$ for data recovery.

Dataset	Metric	$\mathcal{T}=0.1$	$\mathcal{T}=0.3$	$\mathcal{T}=0.5$	$\mathcal{T}=0.7$	$\mathcal{T}=0.9$
Stuart	ACC	**0.9416**	0.8623	0.7286	0.7338	0.7130
	NMI	**0.8319**	0.7826	0.7131	0.7063	0.6438
	ARI	**0.8883**	0.7068	0.5455	0.5499	0.4940
Pbmc	ACC	**0.7297**	0.6877	0.6715	0.6717	0.6555
	NMI	0.6800	**0.7047**	0.6920	0.6996	0.6846
	ARI	0.5913	**0.5957**	0.5798	0.5886	0.5616
Gayoso	ACC	**0.7170**	0.5540	0.5450	0.5445	0.4826
	NMI	**0.6779**	0.6463	0.6560	0.6504	0.4648
	ARI	**0.7311**	0.4976	0.4975	0.4932	0.2975
Smage	ACC	**0.7211**	0.6677	0.5613	0.6251	0.6023
	NMI	0.6103	**0.6215**	0.5757	0.6058	0.5736
	ARI	**0.6246**	0.5434	0.4209	0.5547	0.4704

**Figure 4:**
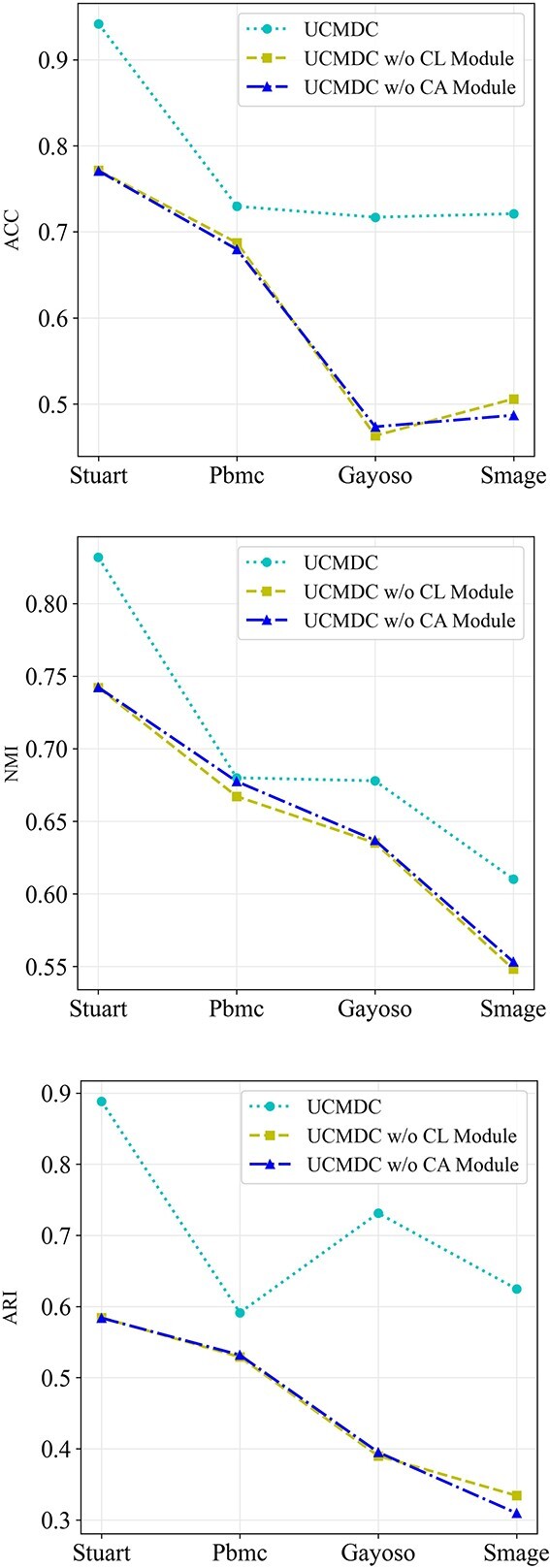
Ablation study on the cross-modal aggregation module and the contrastive learning module. CL stands for the contrastive learning module, while CA represents the cross-modal aggregation module..

In Fig. 4, we presented the performance comparison between these two variants and the complete UCMDC model. The experimental results unequivocally showed that the UCMDC model exhibited superior clustering performance when all modules were retained. This finding not only confirmed the independent value of each module but also highlighted the significant impact of their synergistic operation. When the cross-modal aggregation module was removed, there was a significant decline in model performance, underscoring the critical role of cross-modal aggregation based on global structural in the clustering process. Conversely, removing the contrastive learning module resulted in a marked decrease in clustering performance. The contrastive learning module serves as a directional guide, enabling the model to effectively train on complex data and thereby achieve alignment of clusters across various modes. In conclusion, the results from the ablation experiments are clear: the proposed cross-modal aggregation and contrastive learning modules effectively generated discriminative cell representations and significantly enhanced clustering performance. These modules collectively drive the exceptional performance of the UCMDC model in the field of single-cell clustering.

### Parameter analysis (Q5)

In the UCMDC model, several critical hyperparameters significantly impact the model’s performance. These include the recovery confidence threshold $ \mathcal{T} $, the temperature coefficient $ \mathcal{M} $, and the balance coefficient $ \lambda $, which, respectively, govern the data recovery, contrastive learning, and joint optimization modules.

**Figure 5:**
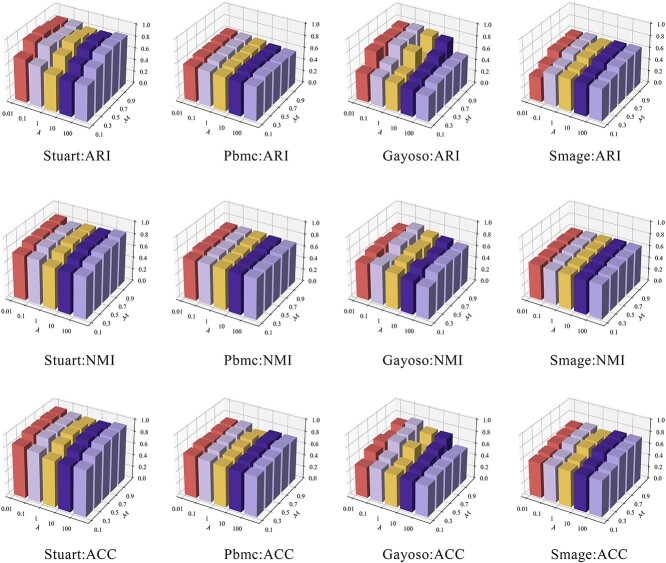
Parameter sensitivity analysis for two hyperparameters: the balance coefficient $\lambda $ and the temperature coefficient $\mathcal{M} $.

This section explores the effects of these three hyperparameters on the clustering outcomes. To accurately determine the optimal data recovery confidence threshold $ \mathcal{T} $, experiments were conducted across four different datasets. We explored $ \mathcal{T} $ at values of $\{0.1, 0.3, 0.5, 0.7, 0.9\}$, with the corresponding clustering scores presented in Table 4. The results suggest that $ \mathcal{T} $ set at 0.1 generally yields the best performance, while $ \mathcal{T} $ at 0.3 occasionally achieves optimal outcomes. Additionally, we investigated the hyperparameters $ \mathcal{M} $ and $ \lambda $, conducting searches within their parameter spaces $\{0.1, 0.3, 0.5, 0.7, 0.9\}$ for $ \mathcal{M} $ and $\{0.01, 0.1, 1, 10, 100\}$ for $ \lambda $, with results illustrated in Fig. 5. The experimental outcomes show that $ \lambda $ has minimal impact on performance, being relatively insensitive in most cases. Conversely, $ \mathcal{M} $ is highly sensitive; improper settings can lead to significant performance degradation, with the model’s sensitivity varying across different datasets. For example, sensitivity to $ \mathcal{M} $ is notable in the Stuart and Gayoso datasets, while it remains unchanged in the Pbmc and Smage datasets. Consequently, since $ \lambda $ shows minimal sensitivity, setting $ \mathcal{M} $ at 0.5 is recommended to achieve optimal clustering performance.

## Conclusion

In this study, we introduce an innovative ’Recover then Aggregate’ deep clustering framework to address the pervasive issue of missing values in single-cell data. Through the synergistic operation of its multiple components, this framework effectively manages single-cell data with missing values and achieves efficient aggregation and optimization, significantly enhancing clustering performance. Initially, the discriminative data recovery module intelligently determines whether to proceed with data recovery by assessing the confidence in zero values, thereby preventing the introduction of external noise that could result from excessive recovery. Subsequently, the cross-view aggregation module utilizes an attention mechanism to construct a global structural relationship matrix and allocates weights to each independent mode, facilitating more effective data integration. Lastly, the contrastive learning optimization module draws samples from the same cluster closer together using contrastive loss and simultaneously optimizes with reconstruction loss, yielding high-quality cellular representations.

Extensive experiments validated the effectiveness of the UCMDC compared to SOTA single-cell clustering methods. Our research not only delivers an effective approach for managing single-cell data with missing values but also offers fresh perspectives and directions for the development of the deep clustering domain. Despite its effectiveness, the UCMDC model has several limitations. The current methodology employs the same recovery confidence algorithm to data from various modalities, failing to adjust for the distinct distributions of each modality, which may result in erroneous data recovery in some instances. Moving forward, we will continue to develop customized recovery confidence algorithms and explore more efficient methods for cross-modal information aggregation, thus further refining the model to address the growing complexity of biological data analysis.

**Key Points**

- **Problem:** we propose a pioneering recovery-then-aggregate clustering framework to fully aggregate cross-modal information through structural alignment. To the best of our knowledge, this is the first attempt to integrate data recovery and aggregation into a unified framework and apply structure-guided contrastive loss from different modalities for cross-modal information aggregation.
- **Algorithm:** we propose a discriminative multi-modal data recovery method and structure-guided aggregation approaches, which improve the quality of single-cell data and achieve better fusion.
- **Evaluation:** we have conducted extensive experiments to validate the effectiveness of UCMDC with state-of-the-art (SOTA) single-cell clustering methods.
